# Intercorrelated variability in blood and hemodynamic biomarkers reveals physiological network in hemodialysis patients

**DOI:** 10.1038/s41598-023-28345-1

**Published:** 2023-01-30

**Authors:** Yuichi Nakazato, Masahiro Shimoyama, Alan A. Cohen, Akihisa Watanabe, Hiroaki Kobayashi, Hirofumi Shimoyama, Hiromi Shimoyama

**Affiliations:** 1 Division of Nephrology, Yuai Nisshin Clinic, Hakuyukai Medical Corporation, 2-1914-6 Nisshin-Cho, Kita-Ku, Saitama, Saitama 331-0823 Japan; 2 Division of Nephrology, Yuai Clinic, Hakuyukai Medical Corporation, Saitama, Japan; 3 PRIMUS Research Group, Department of Family Medicine, University of Sherbrooke, Sherbrooke, QC Canada; 4 Butler Columbia Aging Center, Mailman School of Public Health, Columbia University, New York, NY USA; 5 Department of Environmental Health Sciences, Mailman School of Public Health, Columbia University, New York, NY USA; 6 Division of Nephrology, Yuai Minuma Clinic, Hakuyukai Medical Corporation, Saitama, Japan; 7 Division of Nephrology, Yuai Mihashi Clinic, Hakuyukai Medical Corporation, Saitama, Japan

**Keywords:** Regulatory networks, Haemodialysis, Homeostasis, Diabetes, Epidemiology

## Abstract

Increased intra-individual variability of a variety of biomarkers is generally associated with poor health and reflects physiological dysregulation. Correlations among these biomarker variabilities should then represent interactions among heterogeneous biomarker regulatory systems. Herein, in an attempt to elucidate the network structure of physiological systems, we probed the inter-variability correlations of 22 biomarkers. Time series data on 19 blood-based and 3 hemodynamic biomarkers were collected over a one-year period for 334 hemodialysis patients, and their variabilities were evaluated by coefficients of variation. The network diagram exhibited six clusters in the physiological systems, corresponding to the regulatory domains for metabolism, inflammation, circulation, liver, salt, and protein. These domains were captured as latent factors in exploratory and confirmatory factor analyses (CFA). The 6-factor CFA model indicates that dysregulation in each of the domains manifests itself as increased variability in a specific set of biomarkers. Comparison of a diabetic and non-diabetic group within the cohort by multi-group CFA revealed that the diabetic cohort showed reduced capacities in the metabolism and salt domains and higher variabilities of the biomarkers belonging to these domains. The variability-based network analysis visualizes the concept of homeostasis and could be a valuable tool for exploring both healthy and pathological conditions.

## Introduction

In patients undergoing maintenance hemodialysis (HD) therapy, multiple blood tests are performed on a regular basis to ensure proper management of the patient's physical condition. We have been exploring the implications of intra-individual variabilities in time series data obtained from these patients. For many of the blood biomarkers examined, their variability increased with advancing age, and became accelerated synchronously prior to death^1,2^. Such biomarker variability is often associated with mortality and indicators of ill health^3^. Furthermore, a cumulative index of biomarker variabilities, as estimated by principal component analysis, has been reported to show consistent association with almost all known poor prognostic factors, and is a reliable predictor of frailty and mortality^2,4^. These observations can be reasonably explained by assuming that increased variability in each biomarker represents deterioration of the respective regulatory system, or “dysregulation”. With this reasoning, correlations among the variabilities for different biomarkers would represent a proximity of their regulatory mechanisms. Such inter-variability correlations were found positive for all pairwise combinations of biomarkers, indicating that the regulation of each biomarker cooperates with each other to maintain biological homeostasis^2–4^. Accordingly, we considered that by using the correlations among the variabilities for a sufficient number of biomarkers, it should be possible to get a holistic view of the physiological system.

Similar relationships between intra-individual variability and poor health have also been shown for biological parameters other than blood-based biomarkers. For example, variability in blood pressure (BP)^5,6^, heart rate (HR)^7^, gait^8–10^, body temperature^11^, emotion^12^, and sleep^13^ have been reported to be associated with adverse outcomes. Therefore, whatever the type of biomarker, intra-individual variability may represent its dysregulation.

In this study, we attempted to elucidate the network structure of the physiological systems from the correlations of their biomarker variabilities and examined the impact of underlying pathologies on such variabilities. In that case, incorporating more biomarkers would capture a wider range of the original physiological systems. Accordingly, in addition to the 19 blood biomarkers which we assessed in our previous investigation to estimate the overall physiological dysregulation^4^, three hemodynamic biomarkers: systolic BP (SBP), diastolic BP (DBP), and pulse rate (PR) were newly incorporated into this analysis.

Our objectives were (1) to examine whether variabilities of the BP/PR have comparable properties as other biomarker variabilities and can be treated in the same way; (2) to visualize the physiological network based on multiple biomarker variabilities; and (3) to identify diabetes-specific dysregulations using network analysis.

## Methods

### Patients

A total of 359 patients underwent maintenance HD throughout the one-year data collection period (January 1, 2020, to December 31, 2020) at one of the two participating HD facilities in Saitama-City, Japan. Among them, 341 patients had received more than 100 dialysis treatments and undergone more than 21 of the 24 scheduled blood tests during the aforementioned period. To reduce the impact of the potentially higher variabilities of biomarkers during the HD initiation phase^1^, 7 patients who had been on HD treatment for less than 6 months at the beginning of the data collection period were excluded, and the remaining 334 patients were enrolled in the study. Their age, sex, HD vintage, and body mass index were retrieved from their medical records, and subjects who were receiving antidiabetic drug treatment or having their glycated albumin (GA) levels measured regularly were classified as diabetic, regardless of their GA levels.

This retrospective observational study was conducted with the approval of the institutional ethics committee of Hakuyukai Medical Corporation (approval number: 03-002), in accordance with the principles of the Declaration of Helsinki. All of the patients, who were undergoing HD treatment at the participating facilities in 2020, provided informed consent for use of the clinical records.

### Hemodynamic biomarkers

During each HD session, the SBP, DBP, and PR were measured intermittently using an automatic oscillometric BP monitor built into the dialysis machine, and their records were stored on a connected server computer through a HD management software. The measurements were taken in the sitting or supine position, depending on the patient. The median number of pre-dialysis measurements per patient during the year was 147 (interquartile range: 118–156). The SBP, DBP and PR for each patient were aggregated either monthly or yearly, and their mean levels were abbreviated as SBP-M, DBP-M, and PR-M, respectively. Their variabilities were evaluated as log 10-transformed coefficients of variation (CV = population standard deviation/mean), which were abbreviated as SBP-LCV, DBP-LCV, and PR-LCV, respectively^4^.

### Blood biomarkers

At the participating HD facilities, 19 blood parameters were routinely measured in all the HD patients according to the same protocol. Measurements of the white blood cell (WBC), hemoglobin (Hb), platelet (Plat), albumin (Alb), blood urea nitrogen (BUN), creatinine (Cr), potassium (K), uncorrected calcium (Ca), and phosphate (P) were performed twice a month, and those of the total protein (TP), uric acid (UA), sodium (Na), and chloride (Cl) were measured once a month. In addition, the serum levels of aspartate aminotransferase (AST), alanine aminotransferase (ALT), lactate dehydrogenase (LDH), alkaline phosphatase (ALP), LDL cholesterol (LDL), and HDL cholesterol (HDL) were measured every two months. For diabetic patients, glycated albumin (GA) measurements were undertaken monthly based on the recommendations of the Japanese Society for Dialysis Therapy^14^. The blood samples were taken before the first HD session of the week and analyzed by a single outside laboratory. As with hemodynamic biomarkers, the mean value (M) and variability (LCV) of blood biomarker X were calculated from 1-year data for individual patients and were abbreviated as X-M and X-LCV, respectively. There were no missing M and LCV values for all biomarkers studied in the enrolled patients.

### Statistical analysis

All the statistical analyses were performed in R.3.5.0 (R Core Team, 2018) using the corrplot, gplots, lavaan, psych, qgraph, semPlot, and semTools packages. The results were expressed as the means ± SD, and *P* value of < 0.05 were considered as being indicative of statistical significance. Bivariate correlations between continuous variables in the study items (demographic variables, biomarker levels, and biomarker variability) were assessed by calculating the Pearson or Spearman correlation coefficients according to the distribution of the variables. For combinations containing binary variables, point-biserial correlation coefficients were calculated. The *P*-values were determined by the Welch t-test, Mann–Whitney u-test, or Fisher’s exact test, as appropriate. In the determinations of the correlations among the LCVs, the *P*-values were not adjusted for multiple comparisons since it is evident that almost all the LCVs are correlated with each other and multiple comparison adjustments are only appropriate when there is a risk of overinterpreting a single false positive among many negatives^4^.

The overall relationship among the biomarker variabilities (LCVs) was assessed from their correlation matrix and visualized as a 2-dimensional diagram based on the Fruchterman–Reingold algorithm^15^. It was further assessed by exploratory factor analysis (EFA) and confirmatory factor analysis (CFA). Because the LCVs do not fully satisfy multivariate normality, the CFA was performed with a robust maximum likelihood estimator “MLR” in the lavaan package.

The goodness-of-fit of the factor analysis models was evaluated by determination of the Comparative Fit Index (CFI), Tucker–Lewis Index (TLI), Root Mean Square Error of Approximation (RMSEA) and other relevant parameters. CFI > 0.90 (0.95), TLI > 0.90 (0.95), and RMSEA < 0.08 (0.05) were considered as representing an acceptable (or good) fit of the model^16,17^.

For the two groups comprising the population, a multi-group CFA was applied to determine if the measurement models were comparable and if there were differences in the factor means. In this process, four CFA models with progressively stringent equality constraints (i.e., configural, metric, scalar, and strict invariance models) were compared using the changes in the goodness-of-fit indices^18^. If ΔCFI < − 0.01, ΔTLI < − 0.01, or ΔRMSEA > 0.015 was observed, the compared models were considered as not being equivalent^19,20^. In these estimates of model fit, the chi-square difference tests were also included, but were not applied because they were too sensitive to the sample size^17,19,21^.

## Results

### Patient characteristics

A total of 334 patients were enrolled in this study, and their characteristics and laboratory data are presented in Table 1. The mean patient age was 62.9 ± 11.9 years (range = 28.6–88.4 years), and most of the patients had a long HD vintage (interquartile range = 3.8–15.7 years). Of the 334 participants, 327 were scheduled to receive 3 HD sessions per week, and the remaining 7 were scheduled to receive 2 sessions per week. One hundred and forty-three of the participants had diabetes, of which only two had type 1 diabetes. Of the 22 LCVs examined, only the Na-LCV was rejected for a normal distribution by the Kolmogorov–Smirnov test.

**Table 1:** Characteristics of the study population.

Number of patients	334		
Age (years)	62.9 ± 11.9		
Male/female (n)	255/79		
HD duration (years)	11.1 ± 9.3		
Diabetic/non-diabetic	143/191		
BMI (kg/m^2^)	23.5 ± 5.0		
SBP-M (mmHg)	150.8 ± 18.3	SBP-LCV	− 0.99 ± 0.10
DBP-M (mmHg)	80.0 ± 10.8	DBP-LCV	− 0.99 ± 0.11
PR-M (/min)	75.9 ± 10.0	PR-LCV	− 1.11 ± 0.12
WBC-M (/μl)	6325 ± 1572	WBC-LCV	− 0.93 ± 0.15
Hb-M (g/dl)	11.1 ± 0.7	Hb-LCV	− 1.24 ± 0.18
Plat-M (10^4^/μl)	20.0 ± 6.0	Plat-LCV	− 1.03 ± 0.18
TP-M (g/dl)	6.3 ± 0.4	TP-LCV	− 1.50 ± 0.15
Alb-M (g/dl)	3.6 ± 0.3	Alb-LCV	− 1.36 ± 0.15
AST-M (IU/l)	13.8 ± 6.7	ALT-LCV	− 0.75 ± 0.28
ALT-M (IU/l)	11.6 ± 6.4	AST-LCV	− 0.69 ± 0.26
LDH-M (IU/l)	179 ± 46	LDH-LCV	− 1.11 ± 0.18
ALP-M (IU/l)	224 ± 82	ALP-LCV	− 0.97 ± 0.23
BUN-M (mg/dl)	64.5 ± 12.3	BUN-LCV	− 0.91 ± 0.14
Cr-M (mg/dl)	11.7 ± 2.7	Cr-LCV	− 1.26 ± 0.18
UA-M (mg/dl)	7.2 ± 1.2	UA-LCV	− 1.08 ± 0.17
Na-M (mmol/l)	137.9 ± 2.3	Na-LCV	− 1.96 ± 0.13
K-M (mmol/l)	5.0 ± 0.5	K-LCV	− 1.10 ± 0.15
Cl-M (mmol/l)	103.3 ± 2.7	Cl-LCV	− 1.79 ± 0.14
Ca-M (mg/dl)	8.6 ± 0.5	Ca-LCV	− 1.42 ± 0.18
P-M (mg/dl)	5.3 ± 0.9	P-LCV	− 0.80 ± 0.14
LDL-M (mg/dl)	86.7 ± 26.5	LDL-LCV	− 1.02 ± 0.22
HDL-M (mg/dl)	45.0 ± 13.2	HDL-LCV	− 1.09 ± 0.21
GA-M (%)^a^	19.4 ± 4.0	GA-LCV^a^	− 1.28 ± 0.25

*HD* hemodialysis, *BMI* body mass index. The age and HD duration indicated represent the values as on the first day of the data collection period (January 1, 2020). X-M denotes the annual mean of biomarker X, and X-LCV represents its variability (log-transformed coefficient of variation) calculated from the one-year measurements. Values are means ± SD of the population. ^a^For GA-M and GA-LCV, the values are calculated for diabetic subjects only.

### Pre-dialysis blood pressure, pulse rate, and their variability

**Figure 1:**
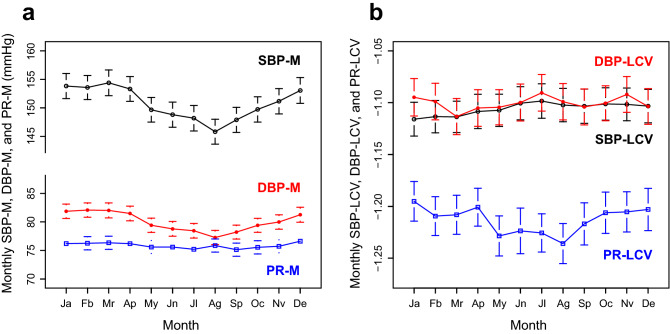
Monthly mean levels and variability of the pre-dialysis blood pressure and pulse rate. *SBP* systolic blood pressure, *DBP* diastolic blood pressure, *PR* pulse rate. The monthly SBP, DBP, and PR measurements were compiled for each patient. The number of eligible patients per month was 331–334. Error bar represents the 95% confidence interval.

To confirm that our hemodynamic data were similar in characteristics to those previously reported, we first examined their seasonal variations. Each patient’s pre-dialysis SBP, DBP, and PR measurements were compiled on a monthly basis, and the seasonal differences were examined. As reported for HD patients^22–25^, the yearly mean SBP in this cohort was high (Table 1), and the monthly mean SBP and DBP were higher in winter and lower in summer (Fig. 1a). Comparison of the values for August and February, as representative values for the summer and winter months by the paired t-test showed a highly significant difference. (SBP-M: 153.6 ± 19.6 vs. 145.8 ± 20.3, *P* < 10^–16^; DBP-M: 82.1 ± 11.6 vs. 77.3 ± 5.9, *P* < 10^–22^). On the other hand, the monthly means of the PR for these months were 76.3 ± 10.8 and 75.9 ± 10.7, and the difference was not significant (*P* = 0.24).

After confirming the seasonal trend of BP levels, we continued to examine the characteristics of BP variability. The monthly SBP/DBP variability did not show any clear seasonal changes (Fig. 1b). There was no significant difference in the values for February and August (SBP-LCV: − 1.11 ± 0.15 vs. − 1.10 ± 0.15, *P* = 0.21; DBP-LCV: − 1.10 ± 0.16 vs. − 1.10 ± 0.16, *P* = 0.96). The PR variability tended to be lower during the summer months, and the difference between the values for February and August was significant (− 1.21 ± 0.17 vs. − 1.24 ± 0.18, *P* = 0.008). The LCV values calculated from all the measurements during the one-year period are presented in Table 1.

### Correlations among the biomarker variabilities

**Figure 2:**
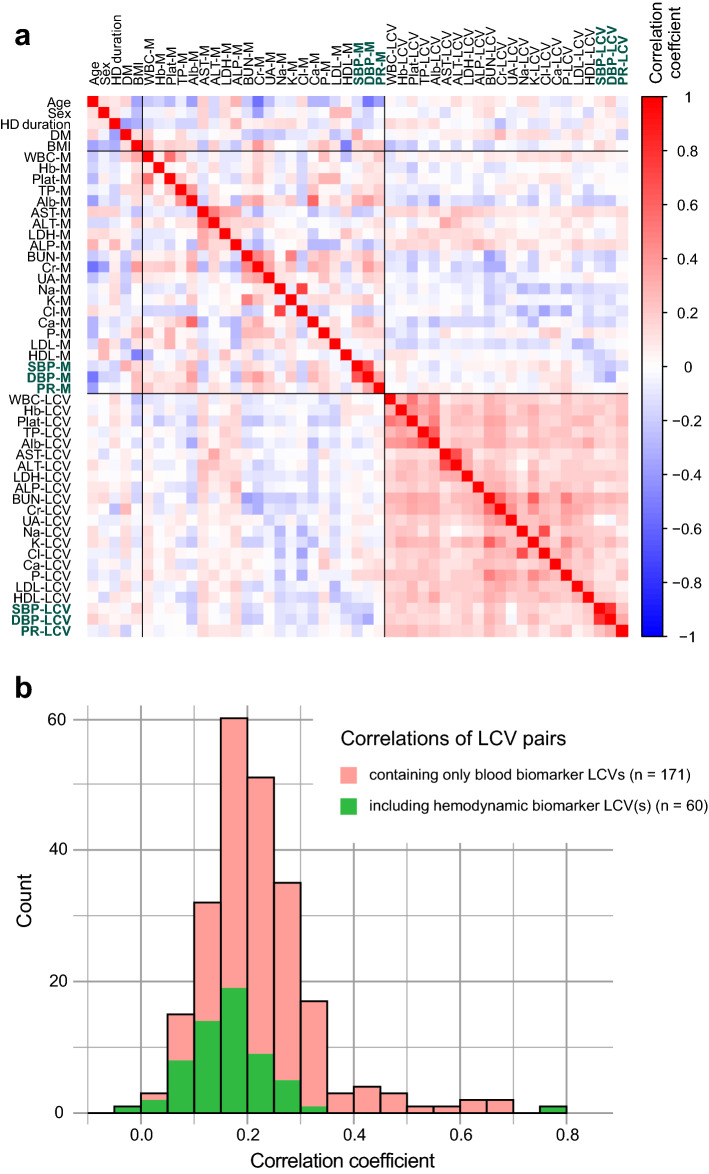
Correlations among the demographic parameters, biomarker levels, and their variabilities. (**a**) The correlation matrix in Supplementary Table S1 is shown as a heatmap. The sign and strength of the correlation coefficient are displayed by the color and its density, respectively. *HD* hemodialysis, *DM* diabetes mellitus, *BMI* body mass index. (**b**) Distribution of the 231 correlation coefficients among the biomarker LCVs is represented as a histogram. Among them, biomarker pairs including at least one hemodynamic biomarker are shown in green.

A total of 22 biomarkers, including 19 blood-based biomarkers and 3 hemodynamic biomarkers, were assessed in this study. Pair-wise correlations among the demographic variables, mean levels of the biomarkers (Ms), and their variabilities (LCVs) were computed, and the entire correlation matrix (52 × 52) is provided as Supplementary Table S1. It is also presented as a heatmap in Fig. 2a. As shown in both the table and figure, the 231 correlation coefficients for the possible pair-wise combinations of the 22 biomarker LCVs were all positive, with one exception (r = − 0.028 between UA-LCV and DBP-LCV, *P* = 0.611). Although the correlations were weak for many combinations, they were significant for 209 of them (90.5%).

Comparison of the correlation coefficients between the two groups of combinations (Fig. 2b), one consisting of only blood biomarkers (171 correlations) and the other including at least one hemodynamic biomarker (60 correlations), revealed generally lower coefficients in the latter group. However, even in the latter group, the correlations were still significant for 49 combinations (81.7%).

In addition, examining the relationship between hemodynamic biomarker *variabilities* and blood biomarker *levels* (Supplementary Table S1) revealed that a higher SBP/DBP variability was associated with lower serum levels of Alb, BUN, Cr, and Ca, and higher serum levels of ALP. Such correlations with prognosis-related factors were also observed for many blood biomarker variabilities. These results lend support to the notion that hemodynamic biomarker variabilities are similar in characteristics to blood biomarker variabilities, and that both represent dysregulation.

### Network structure of physiological regulation

**Figure 3:**
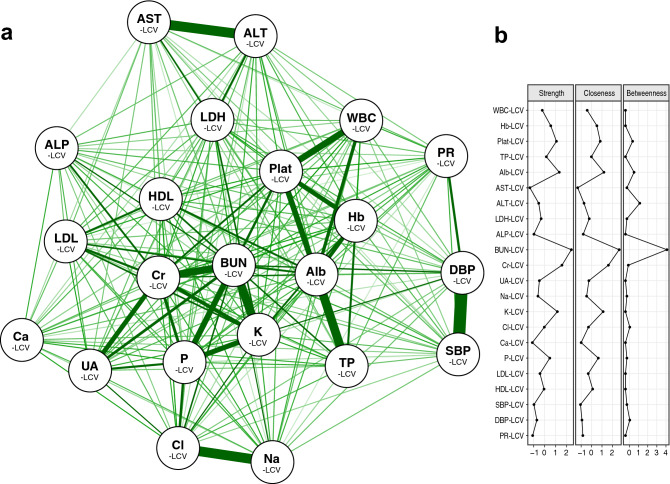
Network structure of physiological regulation. (**a**) The structure of the regulatory systems involving the 22 biomarkers can be visualized as a two-dimensional diagram. Each node labeled a X-LCV represents a regulatory system of biomarker X or its dysregulation. The thickness and relative length of the lines connecting the nodes express the strength of the correlation. Green color denotes a positive correlation. (**b**) Centrality plot for the network depicting the betweenness, closeness, and strength of each node.

Based on the idea that correlations between variabilities in biomarker pairs signify proximity of the two biomarker regulatory systems, we estimated the structure of the physiological network from the correlation matrix of the 22 LCVs. In the network diagram (Fig. 3a), each node originally represents a biomarker dysregulation, but can also be viewed as the biomarker-specific regulatory system itself because the edge connecting 2 nodes designates the proximity of their regulatory systems. These nodes (22 LCVs) formed 5 to 6 clusters, which can be regarded as sub-systems^26^, domains^27,28^, or modules^29^ within the overall physiological system. Because many centrality measures showed very similar patterns for this network, only their representative plots are presented. As depicted in Fig. 3b, BUN-LCV, followed by Alb-LCV, Cr-LCV and K-LCV show high centrality values, suggesting that these regulatory systems may act as the hubs of the physiological network.

### Exploratory factor analyses

To describe the structure of the physiological network in quantitative terms, EFA was conducted for the 22 LCVs. As for the number of factors in the EFA, several estimation methods suggested numbers between 5 and 8. After a close examination of each EFA model with a different number of factors, we selected a six-factor model using maximum likelihood estimation and Oblimin rotation as the most appropriate one. The factor loadings of the model (Table 2) are consistent with the modular structure observed in Fig. 3a and can be visualized as shown in Supplementary Fig. S1. Both diagrams convinced us that the latent factors represent regulatory domains (or sub-systems) for metabolism, inflammation, circulation, liver, salt, and protein. The fit indices of the EFA model are good: χ^2^ (df) = 178.8 (114), χ^2^/df = 1.57; CFI = 0.971; TLI = 0.940; RMSEA = 0.041; Root Mean Square Residual (RMSR) = 0.030.

**Table 2:** Factor loading and variance of the 6-factor EFA model.

	Factor 1	Factor 2	Factor 3	Factor 4	Factor 5	Factor 6
	*Metabolism*	*Circulation*	*Liver*	*Salt*	*Inflammation*	*Protein*
**Factor loading**						
BUN-LCV	**0.82**					
Cr-LCV	**0.61**					
K-LCV	**0.69**					
P-LCV	**0.61**	− 0.11		0.11		
UA-LCV	**0.44**	− 0.20		0.14		
SBP-LCV		**0.78**				
DBP-LCV		**1.00**				
AST-LCV			**0.94**			
ALT-LCV	0.11		**0.72**	− 0.12		
Na-LCV				**0.76**	0.11	
Cl-LCV				**0.88**		
WBC-LCV					**0.54**	
Plat-LCV					**0.84**	
TP-LCV						**0.88**
Alb-LCV					0.31	**0.61**
Hb-LCV					0.34	0.21
LDH-LCV	0.21	0.10	0.27			
ALP-LCV	0.33					
Ca-LCV	0.22			0.13		0.17
LDL-LCV	0.29				0.16	
HDL-LCV	0.14			0.17	0.16	
PR-LCV	0.12	0.25	0.11		0.17	
**Variance explained**						
Proportional	0.12	0.08	0.07	0.07	0.07	0.07
Cumulative	0.12	0.20	0.28	0.35	0.42	0.49

Among the factor loadings, only those with an absolute value of 0.1 or higher are printed, and those with a value of 0.40 or higher are printed in bold.

### Confirmatory factor analysis

**Figure 4:**
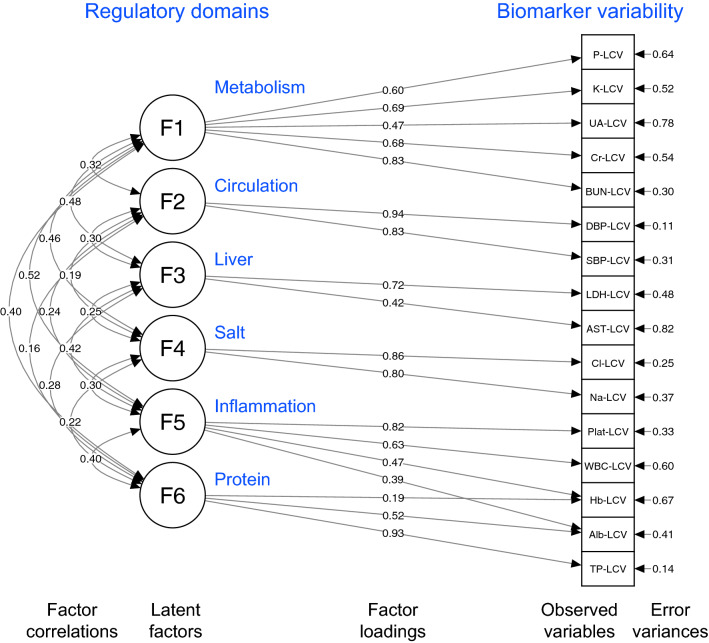
Path diagram for the six-factor CFA model. The model illustrates the associations among regulatory domains (paths with double headed arrows) and between each domain and the observed biomarker variabilities (paths with single-headed arrow). The values are standardized path coefficients.

With reference to the EFA, we developed a simpler CFA model with 6 comparable latent factors and a few cross-loadings (Fig. 4). Except for the χ^2^ test, which is known to be sensitive to the number of samples, the model showed good fit with the alternative fit indices: χ^2^ (df) = 147.6 (87), *P* ≤ 0.001, χ^2^/df = 1.70; CFI = 0.966; TLI = 0.952; SRMR = 0.041; RMSEA = 0.046. The model indicates that the dysfunction of a postulated regulatory domain (= latent factor) increases the variability of a set of biomarkers belonging to it. On the basis of this CFA model, we also constructed second-order and bifactor CFA models (Supplementary Figs. S2 and S3). These additional CFA models showed comparable fit to that of the original CFA model.

### Association of domain-specific dysregulation with clinical parameters

The regulatory capacity of each domain is expected to vary depending on the health status of the patient. Therefore, we estimated factor scores for the six domains and examined their association with various health indicators of the patients (Table 3). Regarding blood biomarkers, lower levels of Alb, BUN, Cr, Na, LDL and higher levels of AST and ALP are associated with dysregulation in most of the domains, while BMI and SBP levels show positive and negative associations that vary by domain. To compare mean factor scores across different groups, it is assumed that the relationship between latent factors and observed variables in each group is approximately equal, i.e., measurement invariance has been established. Therefore, the effects of gender and diabetes were further analyzed using multi-group CFA.

**Table 3:** Correlations between estimated factor scores and study variables.

	F1 *Metabolism*	F2 *Circulation*	F3 *Liver*	F4 *Salt*	F5 *Inflammation*	F6 *Protein*
**Demographics**						
Age	0.086	0.177**	0.078	0.015	0.085	− 0.016
Female/male	0.061	0.075	− 0.023	− 0.046	− 0.007	− 0.058
HD duration	− 0.106	0.030	− 0.009	− 0.063	0.125*	0.077
DM/non-DM	0.181***	0.058	0.102	0.155**	0.048	− 0.012
BMI	− 0.125*	0.110*	− 0.044	− 0.065	− 0.233***	− 0.031
**Biomarker level**						
WBC-M	0.128*	0.069	0.097	0.153**	− 0.010	0.074
Hb-M	− 0.085	− 0.010	− 0.032	0.011	− 0.186***	− 0.045
Plat-M	0.016	0.006	− 0.011	0.096	− 0.064	0.021
TP-M	− 0.005	0.009	0.000	0.015	− 0.074	− 0.096
Alb-M	− 0.197***	− 0.214***	− 0.186***	− 0.087	− 0.267***	− 0.159**
AST-M	0.175**	0.113*	0.258***	0.084	0.240***	0.112*
ALT-M	− 0.005	− 0.011	0.120*	0.036	− 0.047	0.033
LDH-M	0.092	− 0.035	0.183***	0.070	0.151**	0.184***
ALP-M	0.192***	0.161**	0.211***	0.093	0.200***	0.159**
BUN-M	− 0.275***	− 0.218***	− 0.149**	− 0.009	− 0.225***	− 0.101
Cr-M	− 0.265***	− 0.192***	− 0.164**	− 0.027	− 0.225***	− 0.042
UA-M	− 0.149**	0.008	− 0.112*	− 0.040	− 0.073	− 0.006
Na-M	− 0.233***	− 0.115*	− 0.144**	− 0.301***	− 0.136*	0.000
K-M	− 0.146**	− 0.145**	− 0.082	− 0.053	− 0.067	− 0.047
Cl-M	− 0.180***	− 0.071	− 0.131*	− 0.360***	− 0.023	0.033
Ca-M	− 0.192***	− 0.166**	− 0.218***	− 0.073	− 0.217***	− 0.098
P-M	− 0.019	0.061	0.058	0.116*	− 0.137*	− 0.022
LDL-M	− 0.158**	− 0.051	− 0.131*	− 0.163**	− 0.202***	− 0.088
HDL-M	− 0.003	− 0.121*	− 0.019	− 0.030	0.103	− 0.017
GA-M^a^	− 0.005	0.004	0.057	0.048	0.013	0.053
SBP-M	0.075	− 0.206***	0.026	0.139*	− 0.008	− 0.064
DBP-M	− 0.038	− 0.316***	− 0.051	0.048	− 0.051	0.016
PR-M	0.019	− 0.011	0.070	0.023	− 0.024	0.095

*HD* hemodialysis, *DM* diabetes mellitus, *BMI* body mass index. Coefficients printed in boldface indicate P < 0.05. For ^a^GA-M, the coefficients were calculated for diabetic subjects only. Correlations between continuous variables were assessed by Pearson or Spearman correlation coefficients according to the distribution of the variables. For combinations involving dichotomous variables (gender and diabetes status), point biserial correlation coefficients are shown and the *P*-values are determined by Welch t-test. ***P < 0.001, **P < 0.01, *P < 0.05.

### Comparison of diabetic and non-diabetic HD patients

In the multi-group CFA, the diabetic (n = 143) and non-diabetic (n = 191) groups were first analyzed separately using the constructed CFA model (Fig. 4). Then, four models with different equality constraints were compared to see if the measurement structures of the two groups could be considered to be identical (Table 4)^19^. In the increasingly restricted models, changes of the alternative fit indices (ΔCFI, ΔTLI, and ΔRMSEA) were all less than 0.01, and the strict invariance model had the lowest AIC/BIC values. This result justifies that the mean scores of the 6 factors can be compared for both groups in the strict invariance model, in which factor loadings, intercepts, and residual variances are constrained to be equal in the two groups. As shown in Table 5, the diabetic group showed significantly higher factor means for factor 1 (metabolism) and factor 4 (salt), indicating that both domains are more dysregulated in diabetic subjects as compared to non-diabetic subjects.

**Table 4:** Measurement invariance across diabetes status and gender in multi-group CFA.

Models	χ^2^ (df)	χ^2^/df	CFI	TLI	RMSEA	AIC	BIC
**DM/non-DM**							
Configural	240.4 (174)	1.38	0.960	0.945	0.049	− 6485	− 5989
Metric	257.1 (186)	1.38	0.957	0.945	0.049	− 6492	− 6042
Scalar	279.4 (196)	1.43	0.950	0.939	0.052	− 6490	− 6078
Strict	307.2 (212)	1.45	0.945	0.937	0.053	− 6494	− 6144
**Male/female**							
Configural	240.6 (174)	1.38	0.962	0.947	0.049	− 6470	− 5974
Metric	257.7 (186)	1.39	0.959	0.947	0.048	− 6477	− 6027
Scalar	283.2 (196)	1.44	0.951	0.940	0.052	− 6471	− 6059
Strict	333.4 (209)	1.59	0.926	0.915	0.062	− 6447	− 6084

Differences in the fit indices	Δχ^2^ (df)	Δ*P*	ΔCFI	ΔTLI	ΔRMSEA		
**DM/non-DM**							
Metric–configural	17.3 (12)	0.137	− 0.003	0.001	0.000		
Scalar–metric	22.9 (10)	0.011	− 0.007	− 0.006	0.003		
Strict–scalar	24.4 (16)	0.081	− 0.005	− 0.002	0.001		
**Female/male**							
Metric–configural	15.6 (12)	0.210	− 0.002	0.001	0.000		
Scalar–metric	23.9 (10)	0.001	− 0.008	− 0.007	0.003		
Strict–scalar	108.3 (13)	0.000	− 0.025	− 0.025	0.010		

*DM* diabetes mellitus, *χ*^*2*^ chi-square, *df* degree of freedom, *Δχ*^*2*^ chi-square difference, *AIC* Akaike Information Criterion, *BIC* Bayesian Information Criterion.

**Table 5:** Differences in factor means between diabetic and non-diabetic groups and between female and male groups.

Latent factors	DM/non-DM			Female/male		
	Estimate	SE	*P*	Estimate	SE	*P*
F1: Metabolism	0.049	0.014	**< 0.001**	− 0.023	0.018	0.208
F2: Circulation	0.009	0.012	0.449	0.014	0.014	0.295
F3: Liver	0.021	0.019	0.274	0.002	0.013	0.879
F4: Salt	0.034	0.013	**0.011**	0.021	0.019	0.268
F5: Inflammation	0.005	0.012	0.660	0.011	0.022	0.607
F6: Protein	− 0.006	0.017	0.697	− 0.016	0.012	0.180

Significant values are in bold.

Factor means were compared in a strict invariance CFA model between diabetes/non-diabetes and in a scalar invariance CFA model between genders. The non-diabetic group and the male group were used as the reference for comparison, and their factor means were fixed at zero. *SE* standard error.

### Comparison of female and male HD patients

A similar multi-group CFA was conducted between the male and female groups. In this grouping, scalar invariance, rather than strict invariance, was achieved (Table 4), and in the scalar invariance model, the mean factor scores for the six domains did not differ significantly between the gender groups (Table 5). Considering the potential confounding of diabetes and gender, we attempted a multigroup CFA with four groups combining both factors. However, due to the small sample size in each group resulting from the splitting, the analysis could not be completed^30^.

## Discussion

In this study, we analyzed the multivariate structure of the biomarker variabilities in HD patients and obtained a number of novel findings. First, the hemodynamic biomarkers (BP and PR) showed similar patterns to those previously described for blood biomarkers: high variability was a sign of poor health and was correlated with the variabilities of other biomarkers. Second, network analysis showed a clear structure in how the biomarker variabilities correlated with, and presumably influenced, each other. Third, factor analysis identified six key axes of variability in the biomarkers and showed distinct variability profiles in diabetics and non-diabetics. While the overall variability likely remains important, variability in specific domains also appears to contain relevant biological information.

BP variability has been measured in several ways and is categorized into very short-term (beat-to-beat), short-term (along 24 h), mid-term (between days), and long-term (visit-to-visit) variability^5,6^. The BP variability in this study was calculated from all the pre-HD BP measurements made over a 1-year period, and thus can be considered as representing long-term BP variability.

With regard to long-term BP variability, its associations with all-cause and cardiovascular mortality are well documented in the general population^5,6^ as well as in the HD population^31–34^. Such BP variability has also been reported to be larger in the elderly^35,36^ especially those with frailty^37,38^, functional decline^39^, and cognitive impairment^40^.

In contrast to studies on the BP variability, research on HR variability initially focused on very short-term variability. Beat-to-beat HR variability is well known to be negatively associated with the cardiovascular morbidity, mortality, and aging^41,42^. On the other hand, recent studies have shown that long-term HR (or PR) variability is positively associated with all-cause mortality^7,43,44^ suggesting a distinct physiological significance of both types of variations. In any case, variabilities of these basic hemodynamic biomarkers measured at each visit are commonly associated with a poor health status.

Similar to the hemodynamic biomarkers, variabilities in a number of blood-based biomarkers have been shown to be associated with mortality, frailty, and multiple adverse health indicators^2–4,45^. As shown in Fig. 2, the variability levels of the biomarkers are widely cross-correlated, regardless of which biomarker type they belong to. Therefore, all of the biomarker variabilities determined over this timescale seemed to consistently represent physiological dysregulation.

Homeostasis is an essential physiological function for organisms to adapt to environmental changes and adverse stresses, and is sustained by numerous regulatory mechanisms that exist at multiple levels (from molecules to cells, organs, and the body). These regulatory mechanisms are interlinked within cells, between cells, and between organs, and dysregulation of one mechanism can affect the regulation of others through their interactions^29^. Frailty is now recognized as a multi-system physiological dysregulation resulting from such process^46–48^.

To understand the complex interactions within the physiological system, several studies have conducted network analysis using relationship among individual regulatory systems^49^. As a measure of relationship, some studies have employed temporal linkage between different physiological signals^50,51^, while others have used associations among variables representing dysregulation or pathological states^52–54^. In the latter cases, physiologic dysregulation is often assessed by biomarker levels. However, the relationship between biomarker levels and mortality risk can be U-shaped and, moreover, varies depending on the individual's physical condition. In the general population, hypertension, obesity, and hypercholesterolemia increase the mortality risk, but this relationship is known to be reversed in patients with comorbidities such as end stage renal disease and heart failure and in the very elderly (reverse epidemiology)^55–57^. Therefore, assessment of dysregulation by biomarker levels is not straightforward. In this study, we employed biomarker variabilities (LCVs) as measures of dysregulation, which are largely normally distributed and have been reported to be monotonically related to health indicators such as mortality and frailty^2,3,45^. The resulting network diagram, built solely on LCVs, is consistent with our prior knowledge about the regulatory systems, which supports the validity of the LCV-based analysis.

Our CFA model demonstrates the modular structure of the physiological network^29^, in which 6 latent factors are positively correlated with each other (Fig. 4). In the second-order CFA models (Supplementary Fig. S2), these inter-factor correlations are replaced by a higher-order factor, representing the view that systemic dysregulation manifests itself through dysregulation in individual domains. On the other hand, the bifactor CFA model (Supplementary Fig. S3) presents the view that there is a general dysregulation that is independent of dysregulation in individual domains. The comparable fit of these models indicates that the physiological system can be viewed in more than one way and understood as consisting of both general and domain-specific regulation. Such general regulation could be referred to as “allostatic load”^58^, “health status”^52^, or “multisystemic (dys)regulation”^59^.

Subsequent multi-group CFA demonstrated that diabetic patients have greater functional impairment than non-diabetic patients in 2 physiological domains, namely, metabolism and salt. In agreement with the factor structure of the model, the diabetic patients showed higher variabilities of K, Cr, BUN, Na, and Cl (see Supplementary Table S1). Our previous studies, which used datasets from different years (specifically 2002 and 2015–16), also showed similar correlation patterns among blood biomarkers, with higher variability in the same set of biomarkers in the diabetic group^3,4^. These observations on prevalent HD patients suggest that the increased dysregulation in diabetic patients is neither limited to glucose metabolism nor is global, but is related to specific physiological domains.

It has been reported that the levels of various metabolites (lipids, amino acids, glycogen, thiamine, etc.) in organs and blood are altered in patients with type 2 diabetes (T2D), depending on their stage^61,62^. While it has been argued that the early changes may be related to insulin resistance, the specific mechanism remains unknown. The domain we have named metabolism is associated with the regulation of BUN, Cr, P, and UA, suggesting that diabetic HD patients, probably in the late stages of diabetes, have abnormalities in the metabolism of nitrogen-containing compounds such as amino acids. In addition, T2D patients are known to be predisposed to sodium retention from the early stages of the disease^63^. It has been thought that hyperglycemia and the associated diuresis, glucosuria, and hyperinsulinemia cause upregulation of renal glucose transporters and sodium channels, leading to increased renal sodium reabsorption^64,65^. On the other hand, recent studies using ^23^Na magnetic resonance imaging have reported that sodium can be stored in the skin in an osmotically inactive form and that this dermal Na-binding capacity is reduced in T2D patients^60,66^. Our results indicate that in dialysis patients who have already lost renal function, diabetic patients are still more impaired in sodium regulation than non-diabetic patients. In the diabetic HD patients, GA levels do not appear to be associated with dysregulation in any of the domains (Table 3), making it unlikely that hyperglycemia itself is causing sodium dysregulation. In this regard, the decreased dermal sodium-binding capacity seems to be one plausible cause of this dysregulation^64^.

There are several potential limitations to this study. First, although the biomarkers examined in this study are not few, they are far from exhaustive, and the inferred network may only be a part of the real system. As mentioned in the introduction section, if time-series data on other biomarkers, such as gait, sleep, etc., were also available, a more comprehensive physiological network could be constructed. Second, as this study showed, the average BP levels vary seasonally^23^, and the blood parameter levels are also known to have seasonal variations^67,68^. This seasonality may affect the assessment of the variability. It is, however, difficult to extract a seasonal factor from each individual's data. A much larger cohort would be needed for a more refined analysis that takes seasonal factors into account. Third, we performed a multi-group CFA to examine the effects of diabetes and gender on physiological regulatory systems. However, the large sample size required for a multi-group analysis did not allow for a detailed analysis of the interaction of background factors. A larger cohort needs to be prepared for further analysis. Forth, the results obtained in this study are based on data from HD patients in a specific geographic area. To generalize our findings, it would be necessary to confirm them in a separate cohort. The last point that should be mentioned is a problem common to most statistical inferences based on population data. Most statistical studies using aggregate data have been conducted under the assumption that the relationships among observables are approximately the same in each individual of the population (local homogeneity or ergodicity)^59,69,70^. The present study has also estimated individual physiological structures based on statistical inferences derived from inter-individual correlations. However, this generalization from population to individual is not necessarily valid^71^. To strengthen our results, it is necessary to develop a method that can analyze physiological systems on an individual basis.

While the dynamics of various biomarkers have mostly been analyzed separately, it is now clear that they are interrelated. We have shown that the widespread correlations among the biomarker variabilities reflects mutual linkages of the regulatory systems in the body. Network analysis of multifarious biomarker variabilities could be a strong tool for exploring normal and pathological processes in physiological systems.

## Supplementary Information

Supplementary Information 1.

Supplementary Information 2.

## Data Availability

All processed data generated in this study are included in this publication and its Supplementary Information, but the raw data cannot be made openly available to protect the confidentiality of personal information and to comply with the terms of patient consent. Requests related to the raw data should be addressed to the corresponding author.
