# Supplementary material for: Intercorrelated variability in blood and hemodynamic biomarkers reveals physiological network in hemodialysis patients
Source: Sci Rep. 2023 Jan 30;13:1660. doi: 10.1038/s41598-023-28345-1 (PMC9886931; doi:10.1038/s41598-023-28345-1)
Supplement: Supplementary file 1 — Supplementary Information 1. [file 41598_2023_28345_MOESM1_ESM.pdf]

Intercorrelated variability in blood and hemodynamic biomarkers reveals physiological network in hemodialysis patients

Yuichi Nakazato, Masahiro Shimoyama, Alan A. Cohen, Akihisa Watanabe,
Hiroaki Kobayashi, Hirofumi Shimoyama, Hiromi Shimoyama

Supplementary Figures (PDF)

Supplementary Figure S1. Graphic representation of the 6-factor EFA model.
The estimated latent factors (F1 ~ F6) are placed in the inner circle, and the observed variables (biomarker variabilities = LCVs) are placed in the outer circle around the factors. The color and thickness of the edges connecting the two types of nodes indicate the sign and magnitude of the factor loadings, respectively. Green denotes positive, and red denotes negative loading. The fgure was created using R package qgraph 1.6.9 (http://www.jstatsoft.org/v48/i04/).

Supplementary Figure S2. Second-order CFA model.
The values are standardized path coefficients for the items.

Supplementary Figure S3. Bifactor CFA model.
The values are standardized path coefficients for the items. All factors are orthogonal to each other.

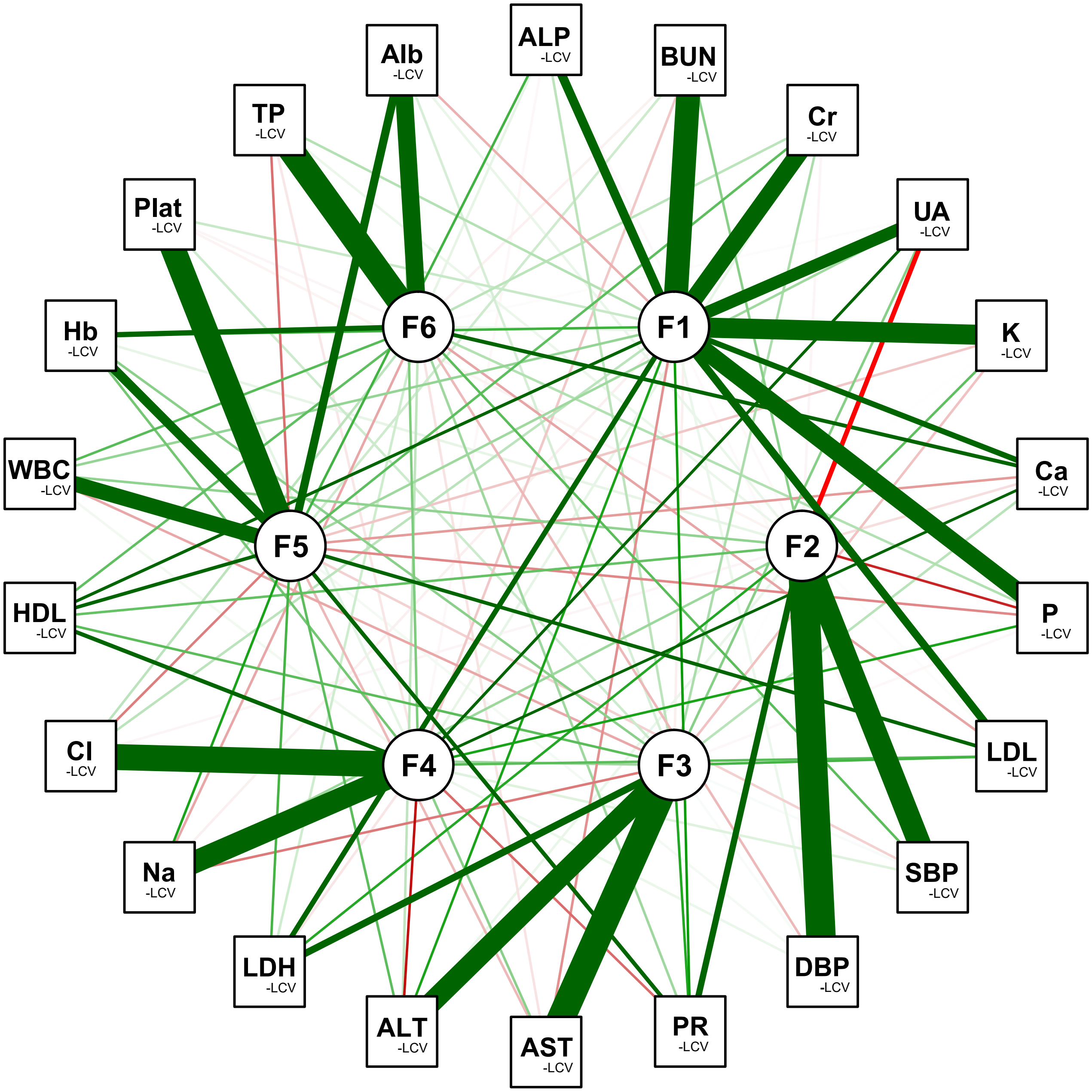

Supplementary Figure S1. Graphic representation of the 6-factor EFA model. The estimated latent factors (F1 ~ F6) are placed in the inner circle, and the observed variables (biomarker variabilities = LCVs) are placed in the outer circle around the factors. The color and thickness of the edges connecting the two types of nodes indicate the sign and magnitude of the factor loadings, respectively. Green denotes positive, and red denotes negative loading. The fgure was created using R package qgraph 1.6.9 (http://www.jstatsoft.org/v48/i04/).

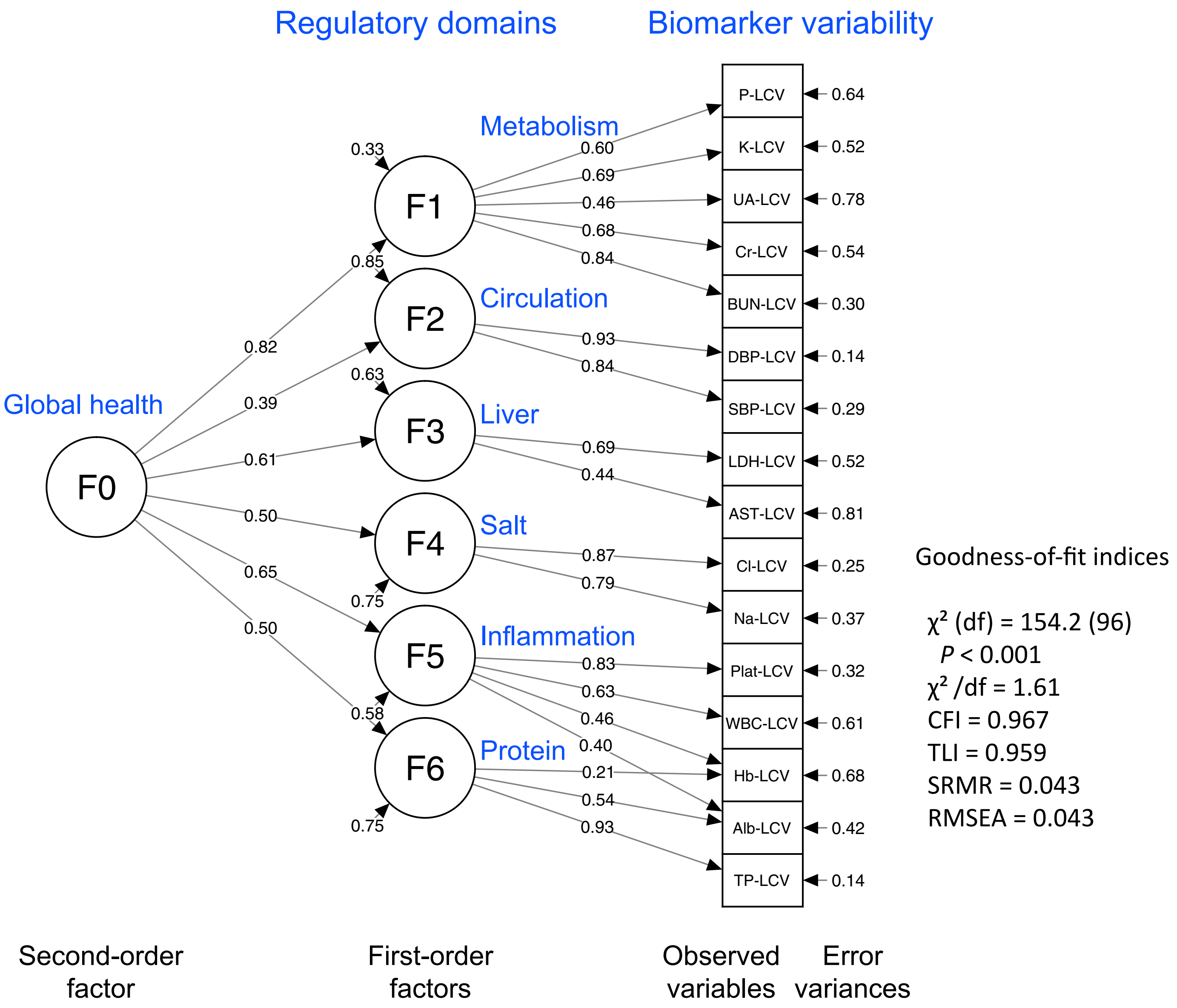

Supplementary Figure S2. Second-order CFA model.
The values are standardized path coefficients for the items.

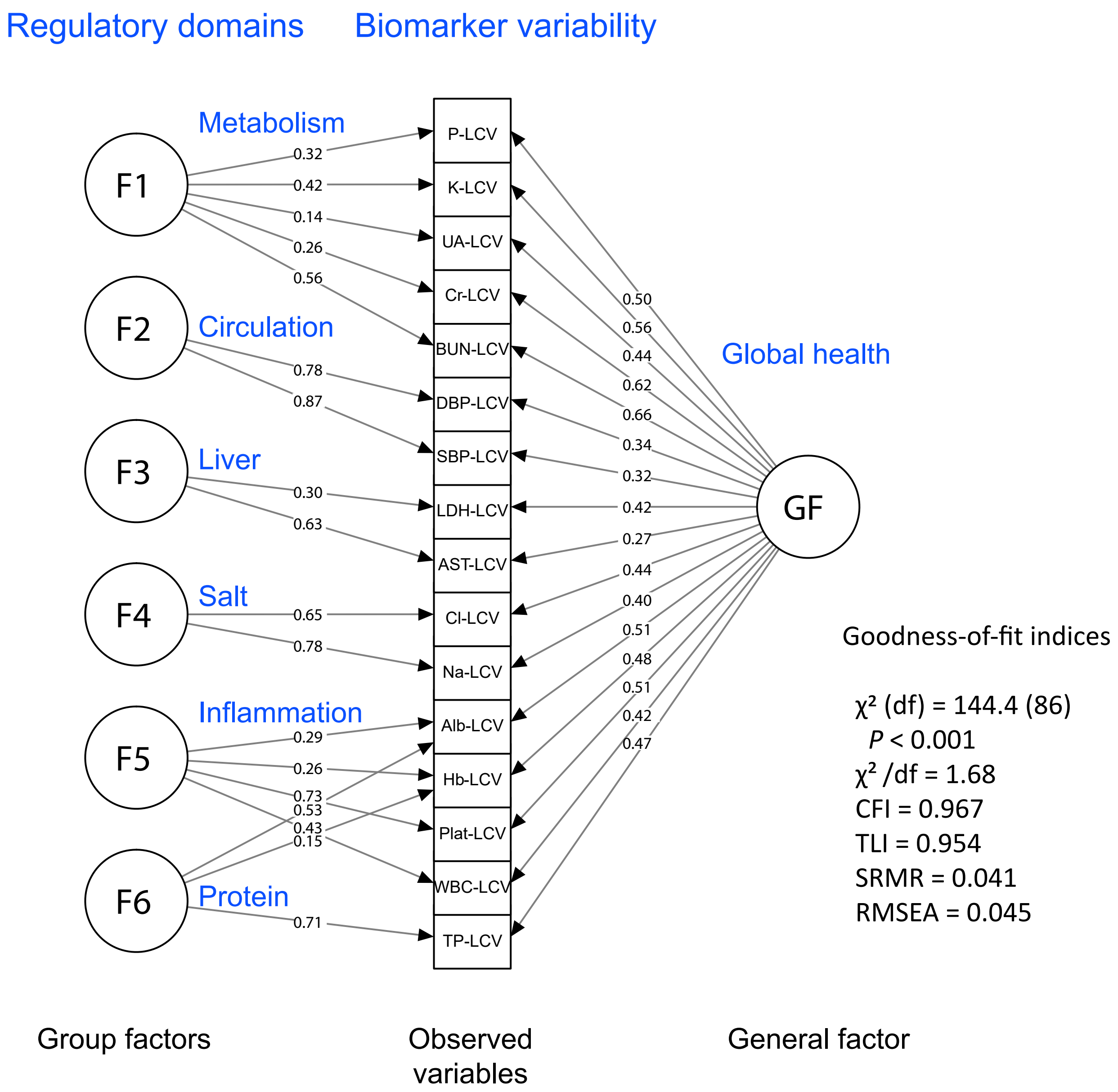

Supplementary Figure S3. Bifactor CFA model.
The values are standardized path coefficients for the items. All factors are orthogonal to each other.
